# Antibacterial and Osteogenic Functionalization of Titanium With Silicon/Copper-Doped High-Energy Shot Peening-Assisted Micro-Arc Oxidation Technique

**DOI:** 10.3389/fbioe.2020.573464

**Published:** 2020-10-08

**Authors:** Xinkun Shen, Wenjia Hu, Linchao Ping, Chongxing Liu, Lili Yao, Zhennan Deng, Gang Wu

**Affiliations:** ^1^School and Hospital of Stomatology, Wenzhou Medical University, Wenzhou, China; ^2^Department of Oral Implantology and Prosthetic Dentistry, Academic Centre for Dentistry Amsterdam (ACTA), Amsterdam Movement Science, University of Amsterdam and Vrije University Amsterdam, Amsterdam, Netherland; ^3^Department of Oral and Maxillofacial Surgary/Pathology, Amsterdam UMC and Academic Centre for Dentistry Amsterdam (ACTA), Vrije Universitetit Amsterdam, Amsterdam Movement Science, Amsterdam, Netherlands

**Keywords:** titanium, high-energy shot peening, micro-arc oxidation, antibacterial, osteogenesis

## Abstract

Antibacterial and osteogenic functionalization of titanium (Ti) implants will greatly expand their clinical indications in immediate implant therapy, accelerate osteointegration, and enhance long-term prognosis. We had recently shown that the high-energy shot peening (HESP)-assisted micro-arc oxidation (MAO) significantly improved the bioactivity and coating stability of Ti-based substrates. In this study, we further functionalized Ti with antibacterial and osteogenic properties by doping silicon (Si) and/or copper (Cu) ions into HESP/MAO-treated coatings. Physicochemical characterization displayed that the doping of Si and Cu in HESP/MAO-treated coatings (Si/Cu-MAO) did not significantly change their surface topography, roughness, crystal structure, coating thickness, bonding strength, and wettability. The results of X-ray photoelectron spectroscopy (XPS) showed that Si and Cu in the Si/Cu-MAO coating was in the form of silicate radical (SiO_3_^2–^) and bivalent copper (Cu^2+^), respectively. The total amounts of Si and Cu were about 13.5 and 5.8 μg/cm^2^, which released about 33.2 and 31.3% within 14 day, respectively. Compared with the control group (MAO), Si doping samples (MAO-Si) significantly increased the cell viability, alkaline phosphatase (ALP) activity, mineralization and osteogenic genes (ALP, collagen I and osteocalcin) expression of MC3T3-E1 cells. Furthermore, the addition of Cu presented good bactericidal property against both *Staphylococcus aureus* and *Streptococcus mutans* (even under the co-culture condition of bacteria and MC3T3-E1 cells): the bacteriostatic rate of both bacteria was over 95%. In conclusion, the novel bioactive Si/Cu-MAO coating with antibacterial and osteogenic properties is a promising functionalization method for orthopedic and dental implants, especially in the immediate implant treatment with an infected socket.

## Introduction

Compared with conventional implantation, immediate implant surgery has been widely used in the restoration of tooth loss due to its advantages of fewer surgical procedures, shorter treatment time and better appearance. However, there are still some contraindications that restrict the clinical promotion of immediate implantation (Koh et al., 2010). At present, socket infection has been considered as one of the most commonly faced complications of immediate implantation (Rass, 2010; Narad et al., 2018). An incomplete pre-implantation curettage (conventional surgical treatment of infected socket before implantation) of infected tissues will leave residual bacteria around the implant which significantly increases the risk of multiple complications and ultimately results in implant failure (Narad et al., 2018). A published meta-analysis has shown that the implant failure rate increase by 116% when the implants are placed directly in a bacterial-infected socket compared with a normal socket (Zhao et al., 2016). Previous study has also shown that low immune resistance in the early post-implantation period will increase the susceptibility of infection, with only 100 bacteria per gram of tissue leading to infection around the implant (Mangram et al., 1999; Zimmerli and Sendi, 2011). The infected bacteria will form a dense biofilm on the surfaces of Ti-based implants in a very short time, which will further block the permeation of host immune cells (such as macrophages) and systemic antibiotics, thereby ensuring the survival of bacteria (Hoffman et al., 2005; Shen et al., 2019a). If not treated in time, infection and secondary inflammation will disrupt the osseointegration of the implant, cause local bone resorption, and finally cause loosening or falling off of implants. Therefore, an effective prevention and treatment of bacterial infection are crucial for the prognosis of long-term implant survivability.

Compared with oral and intravenous antimicrobial agents, a local antibacterial coating on the surface of implants is more preferred by researchers. These locally drug-releasing coatings can allow for low-dose and long-lasting drug therapy, and prevent side effects of the drug on normal tissues or organs (Maher et al., 2018). At present, the active antibacterial agents commonly used in the treatment of Ti-based implants include antibiotics, antimicrobial peptides, and metal ions (Liu et al., 2017; Ghosh et al., 2019; Shen et al., 2019a). However, long-term antibiotics usage may induce drug-resistant bacteria, furthermore, antimicrobial peptides are relatively expensive, so the metal ions-rich antibacterial coatings provide a more extensive application prospect.

In order to improve the bioactivity of titanium (Ti) implants, which are biological inertia, a variety of surface modification strategies have been developed (Szesz et al., 2014; Shen et al., 2019b; Wang et al., 2020). Among them, the bone-like porous coatings with ideal elastic modulus and wear resistance prepared by micro-arc oxidation (MAO) technology are favored and frequently used to prepare commercial Ti-based materials (Szesz et al., 2014; Guo et al., 2020). In our recent work, we have used high-energy shot peening (HESP) pretreatment to significantly improve the comprehensive properties of MAO-treated porous coatings and proved its superior application potential under normal conditions (Shen et al., 2020). However, although the HESP/MAO-prepared porous coatings have been proved to have good bioactivity, the osseointegration of the corresponding implants remains unsatisfactory when exposed to bacterial infection. In the process of MAO treatment, a large number of ion channels will be formed on the surface of Ti, and then suitable ions can be enriched in these porous coatings (Li G. et al., 2019; Shen et al., 2020). Thus, certain metal ions can be selected to further endow the HESP/MAO-treated samples with antibacterial properties. As an essential trace element, copper (Cu) is suitable for the development of antibacterial Ti implants due to its excellent broad-spectrum antibacterial activity against fungus, Gram-positive and Gram-negative bacteria (Macdonald et al., 2011; Xia et al., 2019). Other studies have also reported that application of Cu^2+^ with the appropriate concentration (MC3T3-E1 cells: < 10 μM; human umbilical vein endothelial cells: < 222 μM) can enhance multiple biological properties, which include the promotion of cell proliferation and angiogenesis (Shi et al., 2017; Huang et al., 2019; Li K. et al., 2019).

Furthermore, we add silicon (Si) to the HESP/MAO-prepared coatings to further enhance their osteoinductive potential. It has been reported that Si is located in the active calcification area of bone tissue (Kim et al., 2017) and the appropriate concentration (10–20 μM) of Si can significantly promote the secretion of type I collagen and the differentiation of osteoblasts (Reffitt et al., 2003). By adding Cu and Si to the porous coating concurrently, a new type of multifunctional coating with antibacterial ability and osteoinductive function can be achieved, which is more ideal for preventing implant-related infection and promoting osseointegration.

In this study, the Si/Cu composite porous coatings on HESP-pretreated titanium were successfully constructed for the first time via MAO. The antibacterial effect of Si/Cu doped coatings on *Staphylococcus aureus* (*S. aureus*) and *Streptococcus mutans* (*S. mutans*) were investigated with single and co-culture (bacteria/MC3T3-E1 cells) assays. Furthermore, the spreading, proliferation and differentiation of MC3T3-E1 cells on Si/Cu-added samples were also studied in detail.

## Materials and Methods

### Materials

Ti substrates were obtained from Engineering Research Center for Biomaterials, Sichuan University. Glutaraldehyde, cetylpyridinium chloride, calcium acetate [Ca(CH_3_COO)_2_⋅H_2_O], sodium silicate (Na_2_SiO_3_⋅9H_2_O), and β-glycerophosphate disodium salt (C_3_H_7_Na_2_O_6_P⋅5H_2_O) were purchased from Aladdin Industrial Co. (Shanghai, China). Alizarin red, CCK8, and Live-Dead Cell Staining Kit were provided by Sigma Chemical Co. (MO, United States). LIVE/DEAD^®^ BacLight^TM^ Bacterial Viability kit was received from Molecular Probes (CA, United States). Alkaline Phosphatase (ALP) Assay Kit, Bicinchoninic Acid Assay (BCA) Kit were obtained from Nanjing Jiancheng Biotechnology Institute (Nanjing, China). PrimeScript^®^ RT Reagent Kit and SYBR Premix ExTMTaq II were purchased from Takara Bio Inc. (Kyoto, Japan). Other chemicals were provided by Dingsheng Medical Instrument Reagent Co. (Wenzhou, China).

### Sample Preparation and Characterization

The HESP and MAO technologies were used to treat Ti for preparing target samples according to our previous study (Shen et al., 2020). Briefly, Ti substrates were first polished with gradient sandpapers, cleaned using detergent/alcohol and dried at 60°C. Then, the cleaned samples were treated for 100 s using a HESP device (Shot Peening Machine, Rösler, Germany) under 5 Mpa (pressure) and 0.1 mmA (strength). Glass beads with 0.25–0.3 mm in diameter were used as the shot. After that, HESP-pretreated specimens were further treated with different electrolytes (Tables 1, 2) for 5 min at 480 V using a MAO device developed by Xi’an Technological University. Electrolytes of the target specimens (named as MAO, Si-MAO, Si/Cu-MAO, respectively) evaluated in detail by following cells/bacteria tests.

**TABLE 1:** Compositions of working solution used to construct Si-loaded substrates [MAO, Si1, Si2, and Si3 (Si-MAO)].

	Component contents (moL) in ddH_2_O (1,000 mL)			
Working solution	C_3_H_7_Na_2_ O_6_P⋅5H_2_O	Ca (CH_3_ COO)_2_⋅H_2_O	Na_2_SiO_3_⋅ 9H_2_O	Prepared samples
1	0.05	0.10	0	Si0
2	0.05	0.10	0.02	Si1
3	0.05	0.10	0.04	Si2
4	0.05	0.10	0.08	Si4 (Si-MAO)

**TABLE 2:** Compositions of working solution used to construct Si/Cu-loaded substrates [Si-MAO, Si-Cu1, Si-Cu1 (Si/Cu-MAO), and Si-Cu3].

	Component contents (mol) in ddH_2_O (1,000 mL)				
Working solution	C_3_H_7_Na_2_ O_6_P⋅5H_2_O	Ca (CH_3_ COO)_2_⋅H_2_O	Na_2_SiO_3_⋅ 9H_2_O	Cu(CH_3_COO)_2_⋅ 2H_2_O	Prepared samples
1	0.05	0.10	0	0	MAO
2	0.05	0.10	0.08	0	Si-MAO
3	0.05	0.10	0.08	0.025	Si/Cu1
4	0.05	0.10	0.08	0.05	Si/Cu2 (Si/Cu-MAO)
5	0.05	0.10	0.08	0.075	Si/Cu3

The surface and cross-sectional morphologies of different coatings (MAO, Si-MAO, Si/Cu-MAO) were measured by scanning electron microscopy (SEM, Inspect-F, FEI, United States). The thickness and bonding strength of different coatings were characterized by an eddy current thickness gauge (ED200, Tianxing Research Institute, China) and scratch tester (Revetest Scratch Tester, CSM Instruments, Switzerland), respectively. The surface roughness (Ra), crystalline phase, chemical compositions, chemical states, and water contact angle were further tested by surface roughness meter (Perthometer M1, Mahr, Germany), X-ray diffraction (XRD, X’Pert Pro MPD, Philips, Dutch), energy dispersive spectrometry (EDS, Oxford Instruments, United Kingdom), X-ray photoelectron spectroscopy (XPS, Model PHI 5400, Perkin Elmer, United States), and contact angle measuring instrument (DSA30, Kruss, Germany), respectively.

### Release Profile of Cu^2+^

The specimens were soaked in 6 mL of phosphate buffer saline (PBS) solution at 37°C. After 1, 4, 7, 10, and 14 day, we collected all the soaking solution and re-added another fresh PBS solution (6 mL). In addition, to determine the exact concentration of Si and Cu in the coatings, Si/Cu-MAO samples were soaked in 6 mL of hydrochloric acid (HCl, 3 mol/L) solution for 14 day under oscillatory conditions. The contents of Cu and Si in the PBS or HCl immersion solution were finally measured with an inductively coupled plasma emission spectrometer (ICAP-9000, Jarrell-Ash, United States).

### Bacterial Culture and Morphology

The *S. aureus* (ATCC 6538) and *S. mutans* (ATCC 25175) were provided by the State Key Laboratory of Oral Diseases. After the determination of no other bacterial contamination by Gram stain, a single colony was selected to be inoculated on TSB/LB plates via the streak plate method and continued to culture for 24 h. The individual colonies on the plate were further transferred to liquid medium for culture 10 h before the following bacterial tests.

For detecting the bacterial morphology, two bacteria (1 mL) were cultured on different specimens with a density of 1 × 10^6^ Colony-Forming Units (CFU)/mL. After 24 h, the bacteria attached to the samples were cleaned 3 times with PBS solution, fixed by 2.5% glutaraldehyde for 30 min at 4°C, and then dehydrated with gradient ethanol (30, 50, 75, 85, 95, and 100%). Finally, the bacterial samples were sprayed with gold and observed by SEM.

### Bacteriostasis Rate

The bacteriostasis rate of *S. aureus* and *S. mutans* on different substrates were determined by the attachment film method (referring to GB/T21510-2008). Briefly, 20 μL of bacterial solution (1 × 10^6^ CFU/mL) was added to the surface of different specimens, covered with a cover glass, and cultured for 24 h at 37°C. Bacterial samples were then soaked in 20 mL of PBS solution for 5 min of vortex processing. After diluting 100 times, 0.1 mL of diluted bacteria solution was evenly coated on the agar plate and cultured for another 48 h. Finally, the colony was counted one by one and the bacteriostasis rate was statistically analyzed according to the following formula: Bacteriostasis rate (%) = (Bacterial number in MAO group - Bacterial number in experimental group) / Bacterial number in MAO group × 100%. The experimental group contained Si-MAO and Si/Cu-MAO in this study.

### Live/Dead Staining of Bacteria

One milliliter of *S. aureus* or *S. mutans* (1 × 10^6^ CFU/mL) were seeded on different samples for 24 h. After cleaning 3 times with PBS solution, bacteria were stained using a LIVE/DEAD^®^ BacLight^TM^ Bacterial Viability kit. According to the kit instructions, 100 μL mixture solution of SYTO9 and PI were carefully added to the surface of different specimens and incubated for 15 min. Finally, the stained bacteria were observed using a confocal laser scanning microscope (CLSM, TCS SP2, LEICA, Germany). The final results were analyzed with Leica TCS SP2 Software and presented as 3D images.

### Morphology and Viability of MC3T3-E1 Cells

MC3T3-E1 cells were cultured with low-sugar Dulbecco’s modified Eagle medium (DMEM) supplemented with 10% fetal bovine serum. When the fusion reached 80–90%, cells were digested by 0.25% trypsin and seeded on different substrates at a density of 1 × 10^4^ cells/cm^2^. For morphology observation, cells cultured for 3 day were also fixed by 2.5% glutaraldehyde and dehydrated with gradient ethanol (30, 50, 75, 85, 95, and 100%). The dehydrated cells were further sprayed with gold and observed by SEM. In addition, for viability detection, Cell Counting Kit-8 (CCK8) assay was carried out. Briefly, cells at 1 × 10^4^ cells/cm^2^ were cultured on different substrates for 4 and 7 day. After removing the culture medium, the mixture of CCK-8 solution (30 μL) and DMEM medium (270 μL) was added to each well and incubated for 2 h. 200 μL of the final solution was transferred to a 96-well plate and detected at 450 nm using an enzyme-labeled instrument (Multiskan Spectrum, Thermo Fisher Scientific Inc., United States).

### ALP Activity of MC3T3-E1 Cells

The activity of ALP in MC3T3-E1 cells was determined by spectrophotometry. After culturing for 4 and 7 day on different samples, MC3T3-E1 cells were collected with 0.25% trypsin, creaked by repeated freeze-thaw treatment, and then detected using a ALP Assay Kit. Briefly, 30 μL of sample lysates, phenol standard solution, or double-distilled water were added to a 96-well plate, respectively. Fifty microliter of buffer and 50 μL of matrix solution were then added to each well in turn. After incubation for 15 min at 37°C, 150 μL of color developer was added to each well and detected at 520 nm using an enzyme-labeled instrument. Meanwhile, the concentration of total protein in each group was determined with a BCA Kit at 570 nm to standardize the final ALP activity.

### Mineralization Level of MC3T3-E1 Cells

After culturing for 7 and 14 day on MAO, Si-MAO, and Si/Cu-MAO substrates, MC3T3-E1 cells were fixed by 2.5% glutaraldehyde (30 min) and stained with commercial alizarin red staining solution (60 min). Then, the stained calcium nodule was dissolved using 10% cetylpyridinium chloride and measured at 540 nm.

### Osteogenic Gene Expression

The expression of ALP, collagen I (Col I), and osteocalcin (OCN) genes was measured by real-time fluorescence quantitative PCR (RT-qPCR). After culturing for 7 day, the total RNA of MC3T3-E1 cells was extracted through the Trizol method. The mRNA was then reverse transcribed into complementary deoxyribonucleic acid (cDNA) and determined using a PrimeScript^®^ RT Reagent Kit and SYBR Premix ExTMTaq II, respectively. MAO group was considered the control group in this study. The expression of target genes was standardized with the glyceraldehyde-3-phosphate dehydrogenase (GAPDH) gene. The primers were shown in Table 3.

**TABLE 3:** Real-time polymerase chain reaction primers of osteogenic genes in MC3T3-E1 cells.

Target genes	Primers
ALP	F:5′-AGGGCTGTAAGGACATCGCCTACCA-3′
	R:5′-GACTGCGCCTGGTAGTTGTTGTGAG-3′
COL I	F:5′-CCAGAAGAACTGGTACATCAGCAA-3′
	R:5′-CGCCATACTCGAACTGGAATC-3′
OCN	F:5′-CCTCACACTCCTCGCCCTATTGG-3′
	R:5′-GCTCACACACCTCCCTCCTGG-3′
GAPDH	F:5′-GGCATTGCTCTCAATGACAA-3′
	R:5′-TGTGAGGGAGATGCTCAGTG-3′

### Co-culture of MC3T3-E1 Cells and *S. mutans*

MC3T3-E1 cells (1 × 10^4^ cells/cm^2^) and *S. mutans* (5 × 10^5^ cells/cm^2^) were co-cultured on different specimens for 24 h. For SEM observation, the adherent cells were cleaned 3 times with PBS solution, fixed for 40 min with 2.5% glutaraldehyde, and then dehydrated with gradient ethanol (30, 50, 75, 85, 95, and 100%). The treated samples were sprayed with gold and observed by SEM. Next, to directly observe the living and dead MC3T3-E1 cells on different substrates, the adherent cells were stained with a commercial Live-Dead Cell Staining Kit for another 20 min. The stained cells were visualized using fluorescence microscope (IX71, OLYMPUS, Japan). In addition, the ALP activity and osteogenic genes (ALP and OCN) expression of the co-cultured MC3T3-E1 cells were further determined (refer to 2.8 and 2.10).

### Statistical Analysis

All experiments were independently repeated 3 times. The data was represented by means ± standard deviation. SPSS20.0 packages were used for statistical analysis in this study. The one-way analysis of variance (ANOVA) and Student-Newman-Keuls (SNK) tests were used to determine the differences between different groups. ^∗^*p* < 0.05 (confidence level: 95%) and ^∗∗^*p* < 0.01 (confidence level: 99%) indicated significant differences between groups.

## Results and Discussion

### Concentration Selection of SiO_3_^2–^ and Cu^2+^

**FIGURE 1:**
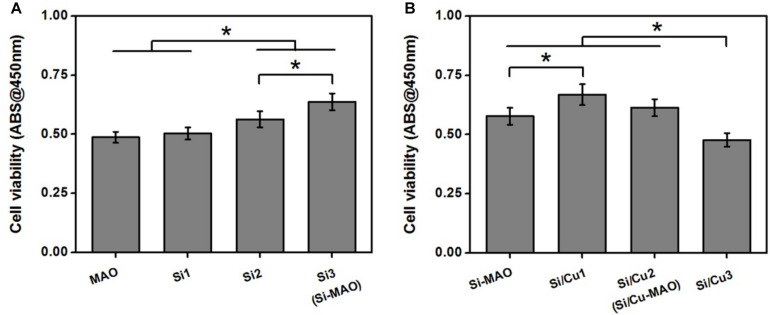
**(A)** Cell viability of MC3T3-E1 cells on Si-loaded specimens [MAO, Si1, Si2, and Si3 (Si-MAO)] at 4 day. **(B)** cell viability of MC3T3-E1 cells on Si/Cu-loaded substrates [Si-MAO, Si-Cu1, Si-Cu1 (Si/Cu-MAO), and Si-Cu3] after 4 day. Error bars represent mean ± SEM for *n* = 6, *p < 0.05.

In order to obtain the best Si/Cu-doped porous structures, the initial concentrations of Na_2_SiO_3_⋅9H_2_O and Cu(CH_3_COO)_2_⋅2H_2_O in the electrolyte were investigated. We selected a concentration range of 0–0.08 moL/L for Na_2_SiO_3_⋅9H_2_O to be used in this study, because higher concentration (>0.08 moL/L) would cause the electrolyte to be cloudy and affect the integrity of target coatings. From Figure 1A, it was found that the ability of the obtained samples to promote osteoblast proliferation increased gradually with the increase of initial SiO_3_^2–^ (Si1 < Si2 < Si3). Therefore, Si3 (also named as Si-MAO) were selected to further prepare the target Si/Cu-doped materials. After adding different concentrations of Cu^2+^ to Si3, the biological activity of target samples was Cu^2+^ concentration-dependent (increasing at first and then decreasing, Figure 1B), which was consistent with previous studies (Cao et al., 2012; Li K. et al., 2019). Since the main reason for adding Cu^2+^ was to endow titanium with excellent antibacterial properties, Si/Cu2 (also named as Si/Cu-MAO) with similar bioactivity to Si-MAO was selected for follow-up studies.

### Physicochemical Characterization

**FIGURE 2:**
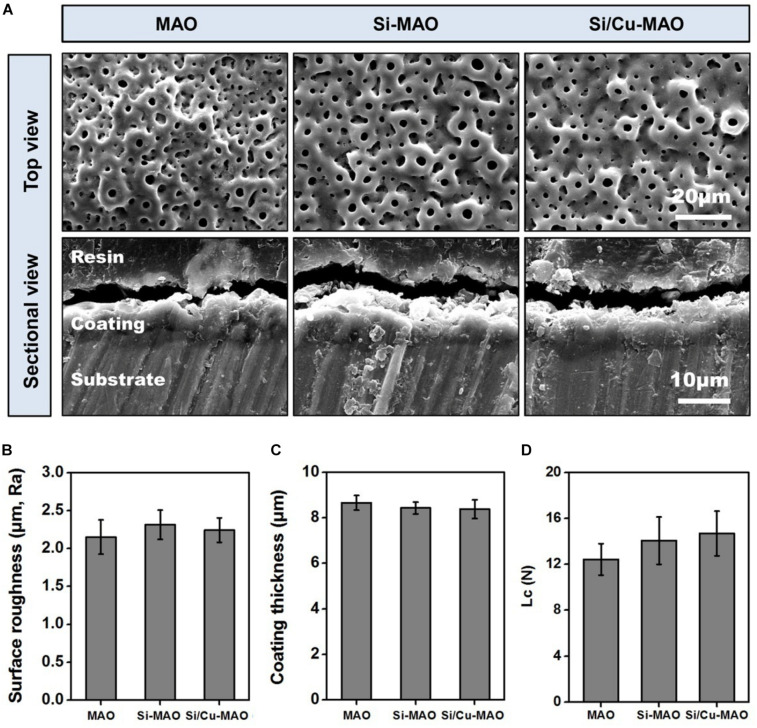
**(A)** SEM images of MAO, Si-MAO, and Si/Cu-MAO samples (top and sectional views); statistics of coating thickness **(B)**, surface roughness **(C)**, and critical loads (Lc, **D**) of MAO, Si-MAO, and Si/Cu-MAO substrates.

The key variables that play a role in affecting the biocompatibility of implant materials include surface morphology, roughness, chemical composition/state, crystalline structure, wettability, and coating stability (Chen et al., 2019; Abaricia et al., 2020). Hence, the in-depth surface characterization of MAO, Si-MAO, and Si/Cu-MAO were conducted. The SEM images (Figure 2A) showed no significant difference in the surface morphology of MAO, Si-MAO, and Si/Cu-MAO, which presented some porous structures (pore size: ∼4 μm). These pores were considered to be the channels for micro-arc discharges during MAO treatment. No obvious cracks were observed on these porous coatings, which was related to the pretreatment of HESP. In our previous study (Shen et al., 2020), we have demonstrated that the formation of coating cracks can be significantly inhibited by the residual compressive stress generated by the HESP treatment. In addition, the cross-sectional SEM images (Figure 2A) and eddy current thickness gauge result (Figure 2B) showed that that the porous coatings of MAO, Si-MAO, and Si/Cu-MAO (about 8.4 μm) were tightly bound to titanium substrates.

EDS results (Table 4) showed that the contents of Ca, P, Ti, and O in MAO samples were about 43.8 ± 0.4, 41.7 ± 0.4, 7.5 ± 0.3, and 7.1 ± 0.2 wt%, respectively. Furthermore, Si was also successfully detected in Si-MAO and Si/Cu-MAO groups (2.4 ± 0.2 and 2.3 ± 0.5 wt%), while Cu was only observed in the latter. The content of Cu (1.4 ± 0.1 wt%) in the Si/Cu-MAO coating was consistent with the range reported in the previous study (Yao et al., 2014).

**TABLE 4:** Statistics of chemical compositions on the surface of MAO, Si-MAO, and Si/Cu-MAO substrates.

	Content of different elements (wt%)					
Samples	Ti	O	P	Ca	Si	Cu
MAO	43.8 ± 0.4	41.7 ± 0.4	7.5 ± 0.3	7.1 ± 0.2	0	0
Si-MAO	41.5 ± 0.6	41.8 ± 0.5	7.1 ± 0.2	7.1 ± 0.3	2.4 ± 0.2	0
Si/Cu-MAO	40.0 ± 0.4	39.3 ± 0.4	6.7 ± 0.2	10.3 ± 0.2	2.3 ± 0.5	1.4 ± 0.1

**FIGURE 3:**
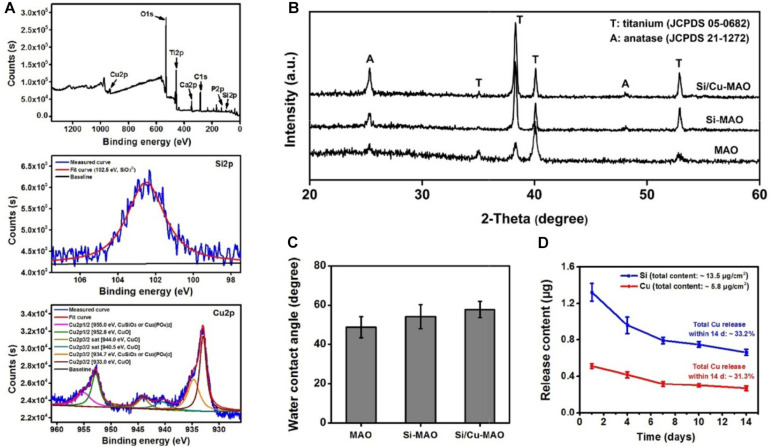
**(A)** XPS pattern and the deconvoluted Si2p/Cu2p of Si/Cu-MAO samples; XRD patterns **(B)** and water contact angles **(C)** images of MAO, Si-MAO, and Si/Cu-MAO substrates; **(D)** release profile of Si and Cu from Si/Cu-MAO specimens at 1, 4, 7, 10, and 14 day.

XPS results (Figure 3A) further proved that Si and Cu were successfully doped in the Si/Cu-MAO coatings. By comparing with the NIST X-ray Photoelectron Spectroscopy Database, the fitting peak of Si2p near 102.5 eV was determined to be silicate radical (SiO_3_^2–^). The characteristic peaks of CuSiO_3_/Cu_3_(PO_4_)_2_ [Cu2p1/2 (955.0 eV) and Cu2p3/2 (934.7 eV)] and CuO [Cu2p1/2 (952.8 eV), Cu2p3/2 (933.0 eV), Cu2p3/2 sat (944.0 eV) and Cu2p3/2 sat (940.5 eV)] were also observed in the fitting curves of Cu2p, indicating that Cu existed in a bivalent form (Cu^2+^).

XRD results (Figure 3B) showed that the three materials have similar patterns except for the difference of peak strength. Only the characteristic peaks of anatase (JCPDS 21-1272) and titanium (JCPDS 05-0682) were observed. This indicates that there was no change in the crystalline structure of MAO coating after the addition of Cu and Si. Furthermore, there was no significant differences observed in the surface roughness (∼2.2 μm, Figure 2C), critical loads (∼14 N, Figure 2D), and water contact angle (∼54°, Figure 3C) among the three materials.

### Release Profile of Cu^2+^

To detect the release behavior of Cu^2+^ and SiO_3_^2–^, Si/Cu-MAO substrates were soaked in PBS/HCl solution for 1, 4, 7, 10, and/or 14 day. Figure 3D showed that the total contents of Cu and Si in the Si/Cu-MAO coatings were about 5.8 and 13.5 μg/cm^2^, respectively. Comparing the amount at different time, Cu^2+^ and SiO_3_^2–^ released on day 1 was the largest (reaching 0.51 ± 0.03 and 1.3 ± 0.10 μg). With the extension of the soaking time, the amount of Cu^2+^ and SiO_3_^2–^ gradually decreased: 0.42 ± 0.03 and 0.96 ± 0.09 μg (4 day), 0.32 ± 0.03 and 0.79 ± 0.04 μg (7 day), 0.30 ± 0.02 and 0.75 ± 0.03 μg (10 day), and 0.27 ± 0.03 (14 day) and 0.66 ± 0.04 μg, respectively. Further statistics showed that the release of Si and Cu accounted for about 33.2 and 31.3% of the total added ions within 14 day. The long-term slow-release property of the Cu^2+^ and SiO_3_^2–^ can endow Si/Cu-MAO specimens with longer antibacterial and osteogenic induction functions.

### Bacteriostasis Rate

*S. aureus* is a common pathogen of implantable infection and has strong pathogenicity. Recent studies have confirmed that *S. aureus* is also a common cause of peri-implantitis (Ulu et al., 2018; Karthikeyan et al., 2019). Moreover, *S. mutans* in the oral cavity is also a common bacterium in inducing peri-implantitis, and can significantly increase the pathogenicity of other bacterial infections (Geremias et al., 2017; Lemos et al., 2019). Therefore, we have selected both *S. aureus* and *S. aureus* to be used for antibacterial studies of our target materials in this study. No significant difference in antibacterial activity was observed between MAO and Si-MAO groups (Figure 3A), which indicated no contribution of Si to antibacterial property. Compared to MAO and Si-MAO, Si/Cu-MAO samples showed a better bacteriostatic effect on *S. aureus* and *S. mutans*, and the antibacterial rate was about 99.1 ± 2.3% and 98.7 ± 3.5%, respectively. This was further confirmed by the Live/Dead staining results as shown in Figure 5. The staining images (Figure 5A) demonstrated the two types of bacteria on the surface of MAO and Si-MAO were stained green (living bacteria), while almost all bacteria on Si/Cu-MAO were stained red (dead bacteria). The dead/total rate (Figure 5B) of bacteria on Si/Cu-MAO substrates were 96.4 ± 3.2% (*S. aureus*) and 94.1 ± 3.9% (*S. mutans*), respectively. These results suggested that Si/Cu-MAO had an excellent broad-spectrum germicidal efficacy against the common oral bacteria.

Previous studies had demonstrated that copper substances/ions could harm the nucleic acid, protein, and lipid of bacteria by changing bacterial membrane potential or inducing excessive active oxygen production (Hong et al., 2012; Tambosi et al., 2018; Zhang et al., 2020). The antibacterial mechanism of Si/Cu-MAO to *S. aureus* and *S. mutans* might be also as follows: firstly, when bacteria attached to Si/Cu-MAO, the doped copper directly destroyed the membrane potential and induced the death of bacteria (contact bacteriostasis; Zhang et al., 2020); secondly, the released Cu^2+^ could bind to the sulfhydryl and amino groups in the bacterial membrane/protein, destroy the energy metabolism and respiratory system of the bacteria, resulting in bacterial death (release bacteriostasis; Tambosi et al., 2018). Therefore, the synergistic effect of contact and release modes endowed Si/Cu-MAO with broad-spectrum antibacterial effect.

### Bacterial Morphology

**FIGURE 4:**
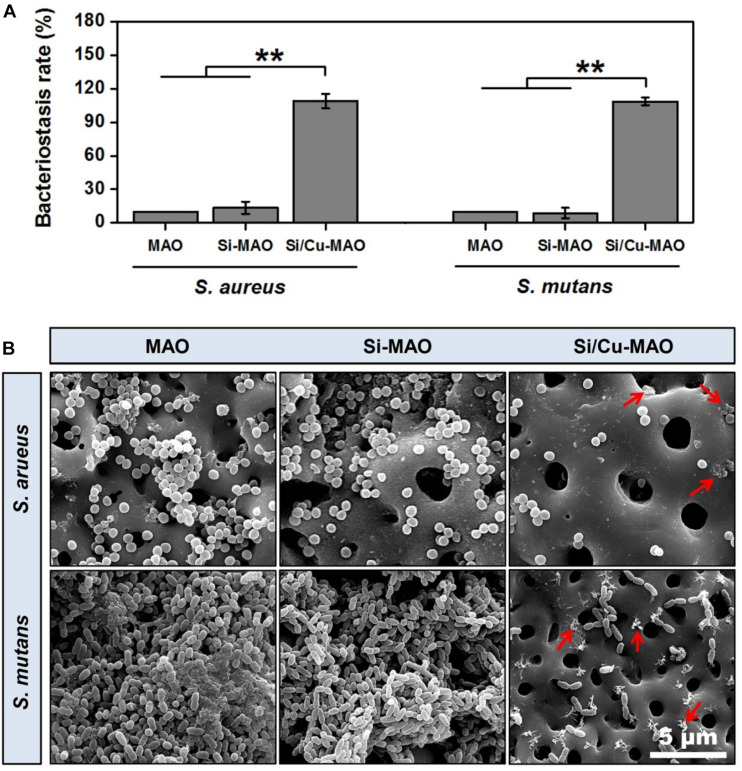
**(A)** Bacteriostasis rate of MAO, Si-MAO, and Si/Cu-MAO samples against *S. aureus* and *S. mutans* at 24 h. Error bars represent mean ± SEM for *n* = 6, **p < 0.01; **(B)** SEM images of two bacteria on MAO, Si-MAO, and Si/Cu-MAO substrates at 24 h (red arrows represent dead bacteria).

**FIGURE 5:**
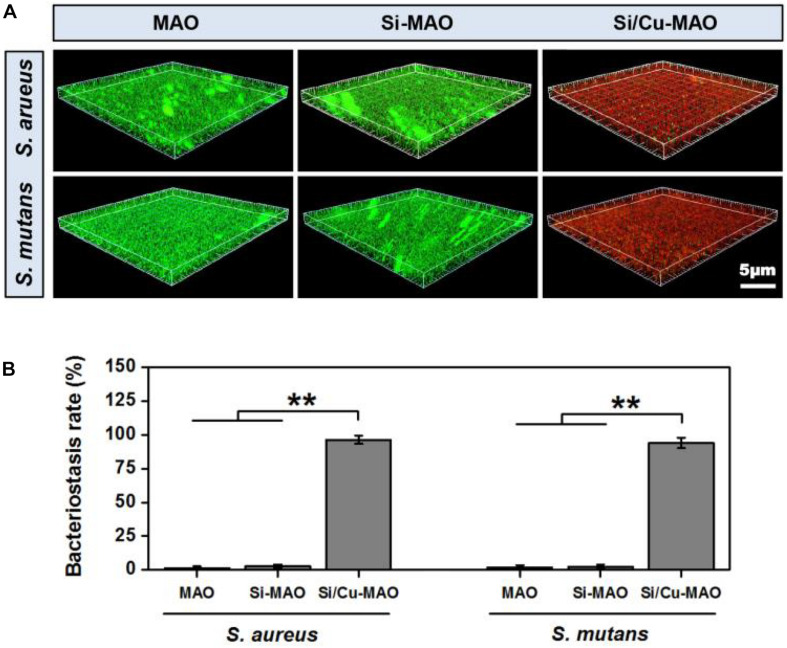
3D fluorescent images (Live/Dead staining, **A**) and dead/total rate **(B)** of two bacteria on MAO, Si-MAO, and Si/Cu-MAO substrates at 24 h. Error bars represent mean ± SEM for *n* = 6, **p < 0.01.

The attachment of *S. aureus* and *S. mutans* to different substrates were observed through SEM, as shown in Figure 4B. A large number of *S. aureus* and *S. mutans* were observed on the surfaces of MAO and Si-MAO, indicating that the addition of SiO_3_^2–^ had no obvious antibacterial effects. Compared with MAO and Si-MAO groups, there was a significant reduction of both bacteria on the surfaces of Si/Cu-MAO samples indicated with red arrows (dead bacteria). This further showed that Si/Cu-MAO could inhibit the early adhesion and proliferation of *S. aureus* and *S. mutans*, which was confirmed by the above results of bacteriostasis rate (Figures 4A, 5).

### Cell Morphology and Viability

**FIGURE 6:**
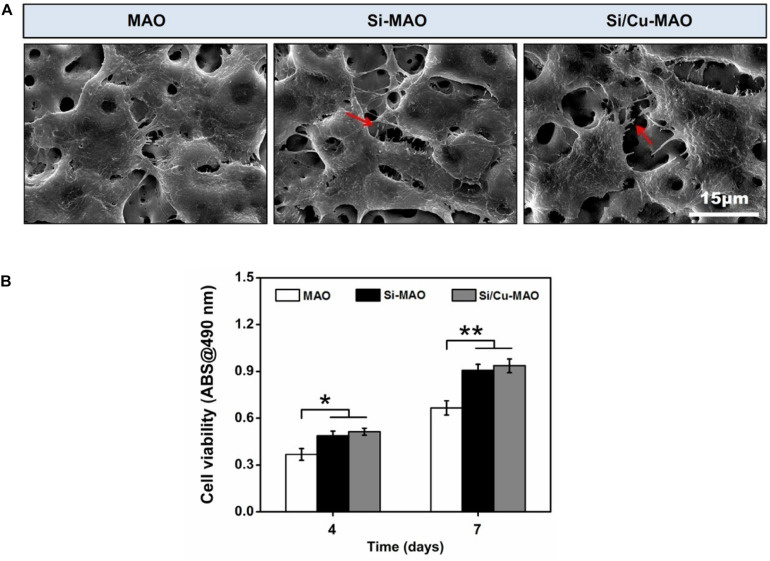
**(A)** SEM images of MC3T3-E1 cells on MAO, Si-MAO, and Si/Cu-MAO samples (red arrows represent pseudopodia of MC3T3-E1 cells); **(B)** cell viability of MC3T3-E1 cells on MAO, Si-MAO, and Si/Cu-MAO substrates at 4 and 7 day. Error bars represent mean ± SEM for *n* = 6, *p < 0.05, **p < 0.01.

To determine the effects of additive SiO_3_^2–^ and Cu^2+^ on cell morphology and viability, SEM observation and CCK-8 measurement were carried out. From SEM results (Figure 6A), it was found that all MC3T3-E1 cells adhered tightly to the surfaces of different materials after 3 day of culture. Compared with the MAO group, the cells on Si-MAO and Si/Cu-MAO groups produced more filamentous pseudopods (red arrows), which extended into the pore structures. The result (Figure 6B) of cell viability also showed that Si-MAO and Si/Cu-MAO significantly (*p* < 0.05 or 0.01) improved the proliferation of MC3T3-E1 cells compared with MAO group after culturing for 4 and 7 day. However, no significant difference of cell viability was observed between Si-MAO and Si/Cu-MAO groups.

In this study, to obtain the best bacteriostatic effect, we chose the highest concentration of Cu^2+^ that MC3T3-E1 cells could tolerate as the parameter of sample preparation (Figure 1B). Thus, the properties of Si-MAO and Si/Cu-MAO in promoting pseudopodia formation and cell proliferation were mainly caused by Si. It had been proved that Si had great potential to cause cell microenvironment alkalization and activate the Ca^2+^ channels to increase the Ca^2+^ influx, thus promoting the early cell adhesion and pseudopods formation (Liu et al., 2013; Wu et al., 2014). In addition, Si could also combine with O_2_ to form silicate with three-dimensional network structures, then adsorb free proteins and interact with cell integrin, and finally promote the adhesion, proliferation and differentiation of osteoblasts (Henstock et al., 2015).

### Cell Differentiation

**FIGURE 7:**
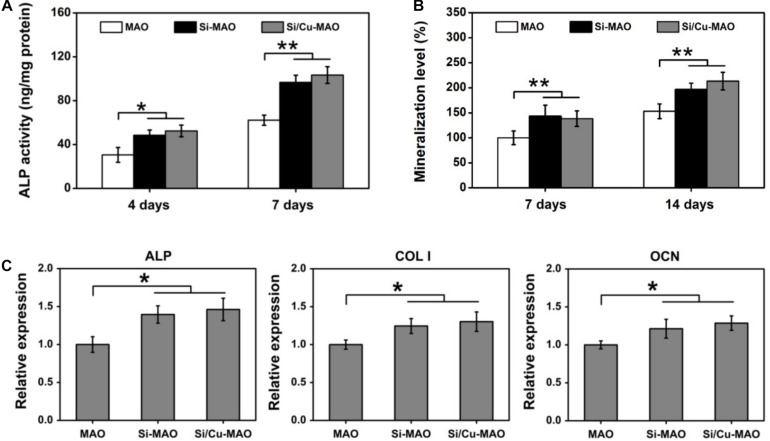
ALP activity **(A)** and mineralization level **(B)** of MC3T3-E1 cells on MAO, Si-MAO, and Si/Cu-MAO substrates at 4, 7, and/or 14 day; **(C)** osteogenic gene expression of MC3T3-E1 cells on MAO, Si-MAO, and Si/Cu-MAO substrates at 7 day. Error bars represent mean ± SEM for *n* = 6, *p < 0.05, **p < 0.01.

As a non-specific phosphomonoesterase, ALP had been proved to greatly hydrolyze inorganic pyrophosphate and increase the formation of hydroxyapatite (Siller and Whyte, 2018; Yu et al., 2018). Thus, ALP activity was frequently selected as a typical early marker of osteoblast differentiation. In addition to ALP, the mineralization level of osteoblasts was also evaluated as a late indicator of osteogenic differentiation in previous researches (Yu et al., 2018; Su et al., 2020). After both 4 and 7 day of culture, the ALP activity of MC3T3-E1 cells in Si-MAO and Si/Cu-MAO groups was significantly (*p* < 0.05 or 0.01) higher than that in MAO group (Figure 7A). The mineralization results (Figure 7B) also confirmed that Si-MAO and Si/Cu-MAO substrates were more effective in promoting osteoblast differentiation. In addition, to further verify the osteoinductive potential of different samples at the molecular level, the expression of ALP, collagen I (Col I) and osteocalcin (OCN) gene was measured after 7 day. The expression trends (Figure 7C) of the three genes were similar to ALP activity and mineralization level: compared with MAO, both Si-MAO and Si/Cu-MAO significantly (*p* < 0.05) enhanced the gene expression of ALP, Col I, and OCN.

The aforementioned results of pseudopodia formation, cell proliferation, ALP activity, mineralization and gene expression showed that Si/Cu-MAO was slightly better than Si-MAO, but there was no significant difference between them. This indicated that the osteogenic effects of Si-MAO and Si/Cu-MAO was mainly attributed to Si rather than Cu. Sun et al. had reported that the Si-doped hydroxyapatite could promote the proliferation and differentiation of osteoblasts via up-regulating MAPK and Wnt signaling pathways (Sun et al., 2018). Dong et al. also claimed that SiO_3_^2–^ could promote the Col I and OCN synthesis of MSCs by activating the BMP-2/Smad1/5/RUNX2 signaling pathway (Dong et al., 2016). Although Cu^2+^ also had superior biocompatibility, they were very dose-dependent (Lin et al., 2016; Li K. et al., 2019). High concentrations of Cu^2+^ would lead to significant cytotoxicity through the oxidative stress pathway (Cao et al., 2012; Li K. et al., 2019). The release of Cu^2+^ from Si/Cu-MAO was about 0.51 μg (∼8 μM) at the first day, which had been close to its safe concentration (∼10 μM) for MC3T3-E1 cells (Li K. et al., 2019). Therefore, compared with Si-MAO, Si/Cu-MAO did not significantly promote the spreading, proliferation and osteogenic differentiation of MC3T3-E1 cells, nor did it show obvious cytotoxicity in this study.

### Co-culture of Bacteria and MC3T3-E1 Cells

**FIGURE 8:**
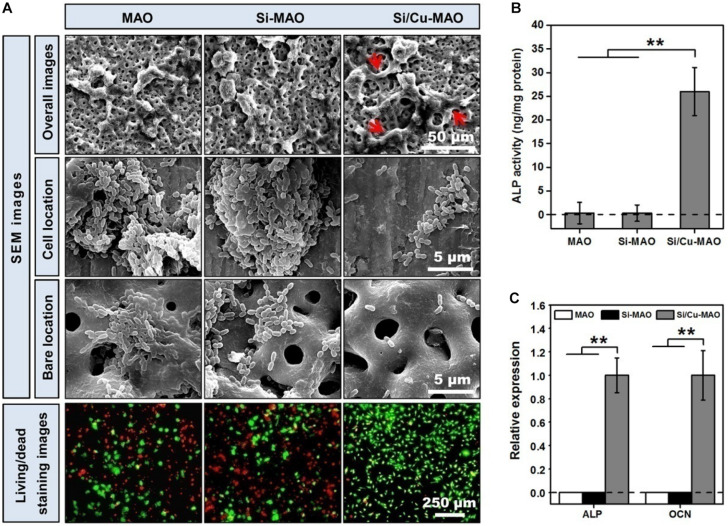
**(A)** SEM (red arrows: spreading MC3T3-E1 cells) and Live/Dead staining (red color: dead MC3T3-E1cells; green color: living MC3T3-E1cells) images of co-cultured MC3T3-E1 cells and *S. mutans* on MAO, Si-MAO, and Si/Cu-MAO substrates at 24 h; ALP activity **(B,C)** osteogenic gene expression of co-cultured MC3T3-E1 cells on MAO, Si-MAO, and Si/Cu-MAO substrates at 7 day. Error bars represent mean ± SEM for *n* = 6, **p < 0.01.

Studies have shown that when bacteria are present, they competitively adhere to the implant surface with the host cells (Subbiahdoss et al., 2011). By forming a dense bacterial membrane or secreting a large amount of toxin, these adherent bacteria induce apoptosis of repair cells and ultimately prevent the formation of new bone (Hoffman et al., 2005; Subbiahdoss et al., 2011; Shen et al., 2019b). The ideal implantation material is expected to inhibit bacterial infection without affecting the normal physiological function of osteoblasts. Therefore, in this study, to further investigate the protective effects of different materials on surface cells in the presence of bacteria, MC3T3-E1 cells and *S. mutans* were further co-cultured for 24 h. The SEM images (Figure 8A) showed that MC3T3-E1 cells on the surfaces of MAO and Si-MAO were round, and many bacteria adhered to the cell surface (cell location images). Many bacteria were also observed in the empty spaces around the cells (bare location images). Compared with the above two groups, the number of bacteria in the cell and bare parts of the Si/Cu-MAO group decreased significantly. The cell spreading of MC3T3-E1 cells (red arrows) on the surface of Si/Cu-MAO group was almost unaffected by bacteria. Live/Dead staining images (Figure 8A) showed that there were a large number of dead cells (red color) on MAO and Si-MAO, and the remaining living cells (green color) also shriveled into a round shape. However, only a small number of cells died on Si/Cu-MAO substrates, indicating that it could ensure the survival of MC3T3-E1 cells effectively in the presence of *S. mutans*. Meanwhile, we found that the round living cells on MAO and Si-MAO appeared to be larger than those in Si/Cu-MAO group, most likely due to the presence of a large number of living bacteria (also appeared green) on the cell surface. Figures 8B,C further showed that only the co-cultured MC3T3-E1 cells on Si/Cu-MAO substrates had significant ALP activity and genes (ALP and OCN) expression. The other two groups showed almost no enzyme activity and gene expression. These results further proved that Si/Cu-MAO substrates had good antibacterial activity and could maintain the normal biological behavior of surface osteoblasts under infection conditions.

## Conclusion

In this study, we prepared three porous coatings (MAO, Si-MAO, and Si/Cu-MAO) with similar surface morphology and crystalline structure by HESP and MAO techniques. Excluding element composition, we have proven that there were no significant differences in the surface roughness (∼2.2 μm), coating thickness (∼8.4 μm) and bonding strength (∼14 N), and water contact angle (∼54°) of the three coatings. Furthermore, *in vitro* cell and bacterial results showed that Si-MAO and Si/Cu-MAO significantly promoted the spreading, proliferation and differentiation of MC3T3-E1 cells compared to the control group (MAO), but only the latter had a better bactericidal effect on both *S. aureus* (antibacterial rate: 99.1 ± 2.3%) and *S. mutans* (antibacterial rate: 98.7 ± 3.5%). All the findings show that the Si/Cu-MAO have excellent comprehensive properties, and has good application prospects in the immediate implant treatment for patients with socket infection.

## Data Availability Statement

All datasets generated for this study are included in the article.

## Author Contributions

XS and CL collated the data and wrote the original draft. WH, LP, and ZD designed the study and carried out the experiments. LY and GW reviewed and edited the manuscript. All authors have read and approved the final submitted manuscript.

## Conflict of Interest

The authors declare that the research was conducted in the absence of any commercial or financial relationships that could be construed as a potential conflict of interest.
